# LIR‐1 educates expanded human NK cells and defines a unique antitumor NK cell subset with potent antibody‐dependent cellular cytotoxicity

**DOI:** 10.1002/cti2.1346

**Published:** 2021-10-05

**Authors:** Caroline Leijonhufvud, Robert Reger, Filip Segerberg, Jakob Theorell, Heinrich Schlums, Yenan T Bryceson, Richard W Childs, Mattias Carlsten

**Affiliations:** ^1^ Department of Medicine Center for Hematology and Regenerative Medicine Karolinska Institutet Stockholm Sweden; ^2^ Cellular and Molecular Therapeutics Branch National Heart, Lung, and Blood Institute National Institutes of Health Bethesda MD USA; ^3^ Oxford Autoimmune Neurology Group Nuffield Department of Clinical Neurosciences University of Oxford Oxford UK; ^4^ Department of Clinical Neuroscience Centre for Molecular Medicine Karolinska Institute Karolinska University Hospital Stockholm Sweden; ^5^ Center for Cell Therapy and Allogeneic Stem Cell Transplantation Karolinska University Hospital Sweden

**Keywords:** antibody‐dependent cellular cytotoxicity, cancer immunotherapy, LIR‐1, NK cell education, NK cells

## Abstract

**Objective:**

KIR and NKG2A receptors educate human NK cells to stay responsive to cells with diminished HLA class I. Here, we addressed whether the HLA class I‐binding receptor LIR‐1 (LILRB1/ILT2/CD85j), which is widely expressed on human NK cells, can mediate education and contribute to antitumor functions of NK cells.

**Methods:**

Healthy donor NK cells either unstimulated, overnight cytokine‐activated or *ex vivo*‐expanded were used to target human cell lines. Phenotype and function were analysed using flow cytometry and ^51^Cr‐release assays.

**Results:**

We found that the inhibitory receptor LIR‐1 can mediate NK cell education under specific conditions. This novel finding was exclusive to expanded NK cells and further characterisation of the cells revealed high expression of granzyme B and DNAM‐1, which both previously have been linked to NK cell education. Corroborating the rheostat education model, LIR‐1 co‐expression with an educating KIR further increased the responsiveness of expanded NK cells. Inversely, antibody masking of LIR‐1 decreased the responsiveness. LIR‐1^+^ expanded NK cells displayed high intrinsic ADCC that, in contrast to KIR and NKG2A, was not inhibited by HLA class I.

**Conclusion:**

These findings identify a unique NK cell subset attractive for adoptive cell therapy to treat cancer. Given that LIR‐1 binds most HLA class I molecules, this subset may be explored in both autologous and allogeneic settings to innately reject HLA class I^‐^ tumor cells as well as HLA class I^+^ target cells when combined with antitumor antibodies. Further studies are warranted to address the potential of this subset *in vivo*.

## Graphical abstract

In this study, we uncover for the first time that the leukocyte immunoglobulin‐like receptor 1 (LIR‐1) inhibitory receptor can mediate natural killer (NK) cell education under specific conditions. This was exclusive to expanded NK cells and associated with potent antibody‐dependent cellular cytotoxicity. The features of LIR‐1^+^ expanded NK cells make them attractive for immunotherapy across human leukocyte antigen class I barriers.

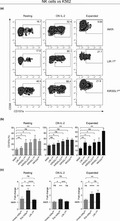

## Introduction

Natural killer (NK) cells are immune cells with the ability to kill virus‐infected and tumor‐transformed cells. Since their discovery, it has been evidenced that NK cells not only have the ability to prevent leukaemic relapse in certain settings of allogeneic stem cell transplantation,^1^ but can also be utilised to treat cancer in settings of adoptive cell transfer.^2^ The latter has been demonstrated using short‐term IL‐2‐activated NK cells, but recent studies have also highlighted the potential of using *ex vivo* expanded NK cells.^3^

In contrast to T cells, NK cells recognise and eliminate cells devoid of HLA class I. This phenomenon is referred to as missing‐self recognition^4^ and is mainly controlled by the inhibitory HLA class I‐binding receptors KIR and NKG2A.^5^ However, in the absence of inhibition, signalling from activating receptors such as NKp30, NKp44, NKp46, NKG2D, 2B4 and DNAM‐1 is required to trigger NK cell cytotoxicity.^6^ In resting NK cells, it has been shown that simultaneous signalling from at least two activating receptors is required to trigger proper NK cell degranulation and subsequent target cell killing.^7^ An exception to this is the Fc‐binding receptor CD16 that alone can trigger NK cell degranulation via antibody‐dependent cellular cytotoxicity (ADCC).^7^

In order to reach and maintain their full cytotoxic potential, NK cells undergo a functional training process referred to as NK cell education.^5^, ^8^, ^9^ This is a dynamic process in which the responsiveness to missing‐self of a given NK cell is tuned by the strength of interactions between its KIR and/or NKG2A inhibitory NK cell receptors (iNKR) and their cognate HLA class I ligands.^10^ NK cells lacking these inhibitory receptors (iNKR^‐^), or NK cells expressing a KIR to which the cognate HLA class I ligand is absent, remain hyporesponsive. In contrast, NK cells that express NKG2A and/or one or several KIRs to which the cognate ligands are present become educated, and as such are potent killers in response to missing‐self. Importantly, NK cells co‐expressing several KIRs to which the corresponding ligands are present exhibit higher responsiveness than NK cells only expressing one of these KIRs.^11^ This process is referred to as co‐education. Although extensive research has been carried out to elucidate the exact mechanisms governing NK cell education, the signalling involved still remains largely unknown. Yet, investigators have associated higher expression of DNAM‐1 and granzyme B to educated NK cell subsets.^12^, ^13^, ^14^, ^15^ Furthermore, educated NK cells have a secretory lysosomal composition that is different to that of uneducated NK cells.^14^

NKG2A and KIRs display specific binding to certain groups of HLA class I molecules.^16^, ^17^ In contrast, the HLA class I‐binding inhibitory receptor leukocyte immunoglobulin‐like receptor 1 (LIR‐1; LILRB1/ILT2/CD85j) has broader specificity as it binds the conserved β_2_m and α3 regions of both classical and non‐classical HLA I molecules.^18^, ^19^, ^20^ Although it binds the majority of HLA class I molecules, the affinity is considered generally weaker than that of KIR and NKG2A receptors.^21^ Among HLA class I, LIR‐1 binds with the highest affinity to the non‐classical molecule HLA‐G,^22^ whose role has mainly been highlighted in maternal–fetal tolerance.^23^ The weak affinity for HLA class I and low responsiveness to missing‐self by freshly isolated LIR‐1^+^ NK cells^11^, ^24^ may explain why this receptor has not been broadly discussed with respect to NK cell targeting of tumor cells. Nonetheless, because it can signal via similar pathways such as NKG2A and inhibitory KIRs^25^ and is expressed on 5–80% of NK cells,^26^ LIR‐1 remains of interest with respect to its potential role in NK cell education and immunotherapy of cancer.

Here, we reveal for the first time that LIR‐1 educates *ex vivo* expanded, but not freshly isolated or short‐term IL‐2‐activated, NK cells. This observation was associated with increased DNAM‐1 and granzyme B expression within the LIR‐1^+^ expanded NK cell subset. Whereas LIR‐1 was able to co‐educate KIR‐educated expanded NK cells, antibody‐mediated blockade of LIR‐1 specifically decreased the responsiveness of LIR‐1^+^ expanded NK cells. Further characterisation uncovered that LIR‐1^+^ expanded NK cells had potent ADCC capacity. Unlike the inhibitory KIR and NKG2A receptors, activation via ADCC was able to override LIR‐1‐mediated inhibition of expanded NK cells. Our data thus indicate that LIR‐1^+^ expanded NK cells represent a subset with unique reactivity and have the potential to be utilised in both autologous and allogeneic adoptive NK cell protocols to safely and selectively target malignant cells in cancer patients.

## Results

### LIR‐1^+^ expanded NK cells show high responsiveness to K562 cells and exhibit co‐education features when expressed with educating KIRs

**Figure 1:**
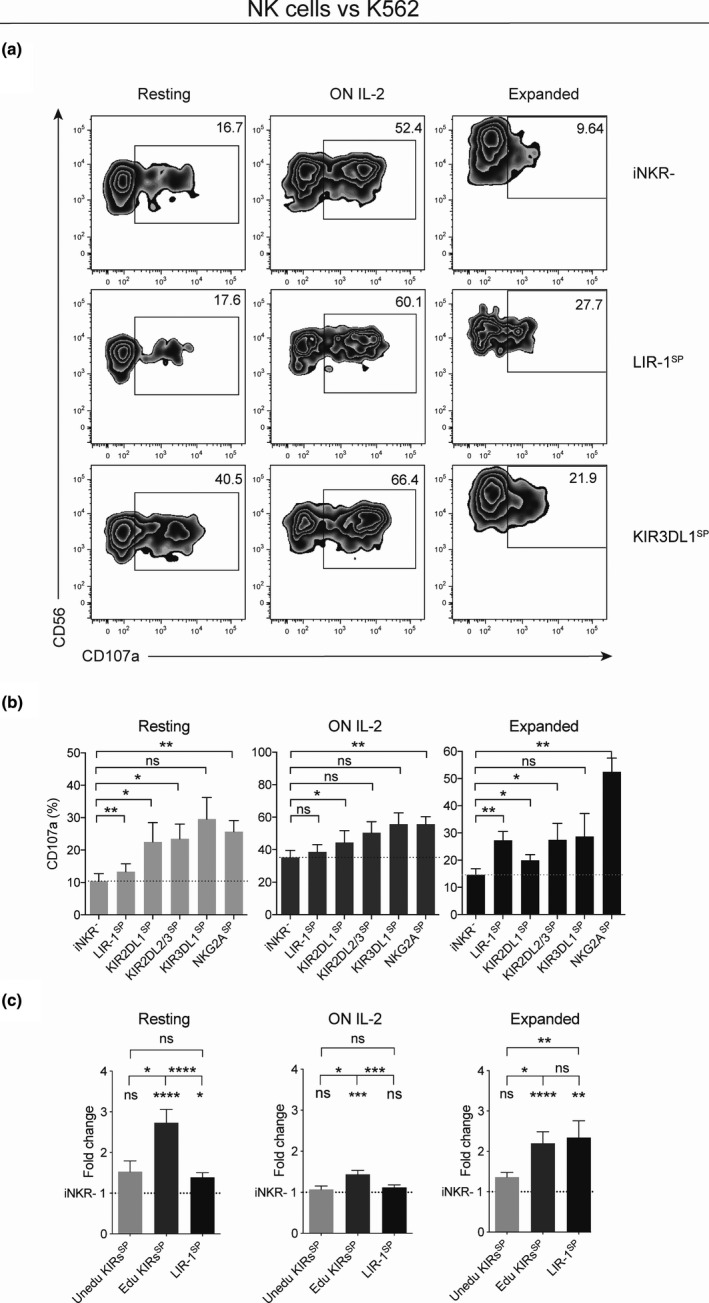
LIR‐1^+^ expanded NK cells show increased responsiveness to K562 cells compared with their resting and overnight IL‐2‐activated counterparts. NK cell degranulation as measured by CD107a cell surface expression on resting, overnight (ON) IL‐2‐activated and expanded NK cells from KIR haplotype A/A donors following co‐culture with K562 cells. **(a)** Zebra plots showing degranulation by selected NK cell subsets from one representative donor educated on the KIR3DL1 ligand HLA‐Bw4. **(b)** Pooled data comparing degranulation by NK cell subsets either lacking all of the investigated inhibitory receptors (iNKR^‐^) or single positive (SP) for LIR‐1, educated KIR or educated NKG2A (iNKR^‐^, *n* = 10; LIR‐1^SP^, *n* = 10; KIR2DL1^SP^, *n* = 7; KIR2DL2/3^SP^, *n* = 7; KIR3DL1^SP^, *n* = 4; and NKG2A^SP^, *n* = 10). Paired analysis was performed using the Wilcoxon matched‐pairs signed‐rank test. **(c)** Pooled data comparing fold change in degranulation by NK cell SP‐uneducated KIR (*n* = 10), NK cell SP‐educated KIR (*n* = 18) and LIR‐1 (*n* = 10) subsets over its individual matched iNKR^‐^ subset. Paired analysis was performed using the Wilcoxon signed‐rank test between LIR‐1^SP^ and the matched groups of uneducated and educated KIR^SP^, respectively, while unmatched analysis was performed between uneducated and educated KIR^SP^ groups using the Mann–Whitney *U*‐test. Individual groups were compared with iNKR^‐^ using the Wilcoxon signed‐rank test and comparing with a hypothetical value of 1. The education status of each KIR was determined on receptor expression and genomic presence of cognate KIR ligand. Bars show mean, and error bars represent SEM.

The responsiveness of non‐cytokine‐activated resting, overnight (ON) IL‐2‐activated and expanded NK cells was addressed by assessing degranulation levels following co‐cultures with the HLA class I^‐^ gold standard NK cell target cell line K562. To understand the potential of the HLA class I‐binding inhibitory KIR, NKG2A and LIR‐1 receptors in mediating education, NK cells from KIR ligand‐genotyped KIR haplotype A/A donors were used. As demonstrated in Figure 1, expanded NK cells expressing LIR‐1 while lacking KIR and NKG2A expression (LIR‐1 single‐positive (LIR‐1^SP^) NK cells) showed significantly higher degranulation levels than iNKR^‐^ NK cells, which represents a hyporesponsive subset as it lacks receptors mediating NK cell education. This observation among expanded NK cells was in sharp contrast to that of resting and ON IL‐2‐activated NK cells (Figure 1a and b), where LIR‐1^SP^ NK cells were hyporesponsive compared with KIR‐educated NK cells. The responsiveness to K562 cells among LIR‐1^SP^ expanded NK cells was similar to that of KIR‐educated expanded NK cells (Figure 1c). The observation that LIR‐1^SP^ expanded NK cells had increased responsiveness compared with iNKR^‐^ expanded NK cells was confirmed in Chromium‐release assays using FACS‐sorted NK cell subsets (Supplementary figure 1). Finally, as the observation was recapitulated using feeder‐free expansion protocols (Supplementary figure 2) and NK cells from cytomegalovirus (CMV) sero‐negative individuals (Supplementary figure 3), the possibility that the phenomenon was feeder cell‐dependent or influenced by the CMV status of the donor and thereby potentially linked to adaptive NK cells that generally express LIR‐1^27^, ^28^ was ruled out.

**Figure 2:**
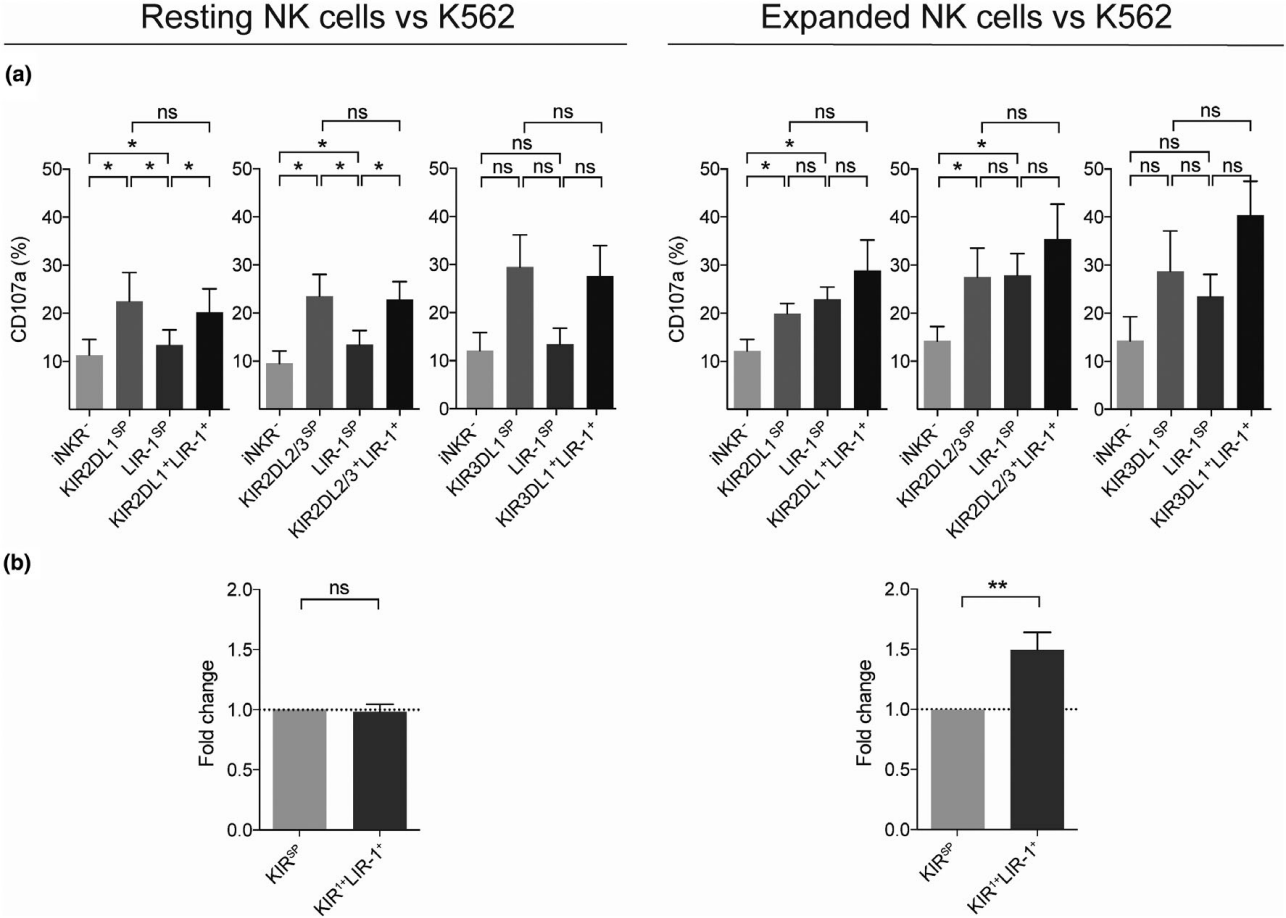
LIR‐1 co‐expression with an educating KIR receptor increases the responsiveness of expanded NK cells. NK cell degranulation as measured by CD107a cell surface expression on resting and expanded NK cells from KIR haplotype A/A donors following co‐culture with K562 cells. **(a)** Gated on NK cell subsets co‐expressing one educated KIR and LIR‐1 compared with NK cell subsets expressing only one educated KIR or the LIR‐1 receptor or cells lacking inhibitory receptors (iNKR^‐^) (KIR2DL1 graph, *n* = 7; KIR2DL2/3 graph, *n* = 7; and KIR3DL1 graph, *n* = 4). Paired analysis was performed using the Wilcoxon matched‐pairs signed‐rank test. **(b)** Pooled data showing fold change degranulation by educated NK cells co‐expressing LIR‐1 with an educating KIR compared with the corresponding NK cell subset only expressing the educating KIR (*n* = 18). KIR^1+^LIR‐1^+^ cells were compared with KIR^SP^ using the Wilcoxon signed‐rank test and comparing with a hypothetical value of 1. Bars show mean, and error bars represent SEM.

Next, the potential for LIR‐1 to mediate co‐education of NK cells, as previously reported for KIR receptors,^11^ was addressed. By comparing the degranulation levels of NK cells expressing an educating KIR to that of NK cells co‐expressing the same KIR together with LIR‐1, it was revealed that co‐expression on expanded NK cells, but not on resting NK cells, resulted in increased responsiveness compared with that of NK cell subsets expressing only one of the two receptors alone (Figure 2a). By pooling data from the KIR2DL1, KIR2DL2/3 and KIR3DL1 NK cell subsets co‐expressing LIR‐1 and comparing that with KIR^SP^ NK cell subsets, it was statistically confirmed that LIR‐1 on expanded but not resting NK cells was able to co‐educate KIR^+^ NK cells (Figure 2b).

Taken together, these data demonstrate that the HLA class I‐binding receptor LIR‐1 is associated with increased responsiveness of expanded NK cells, indicating that it may mediate NK cell education.

### Expanded LIR‐1^SP^ NK cells express high levels of key activating NK cell receptors and granzyme B and no longer have a phenotype overlapping with uneducated hyporesponsive NK cells

*Ex vivo* expansion of human NK cells results in increased expression of several activation receptors and a higher proportion of NKG2A^+^ versus KIR^+^ NK cells.^29^ Therefore, we next analysed whether alterations to the expression pattern of key activation/cytotoxicity molecules could be linked to the increased responsiveness observed among LIR‐1^+^ expanded NK cells. To this end, we used both KIR haplotype A/A and B/x donors. The KIR^SP^ subsets were grouped as educated or uneducated based on the genomic presence of inhibitory KIR and the presence/absence of its cognate KIR ligand.

**Figure 3:**
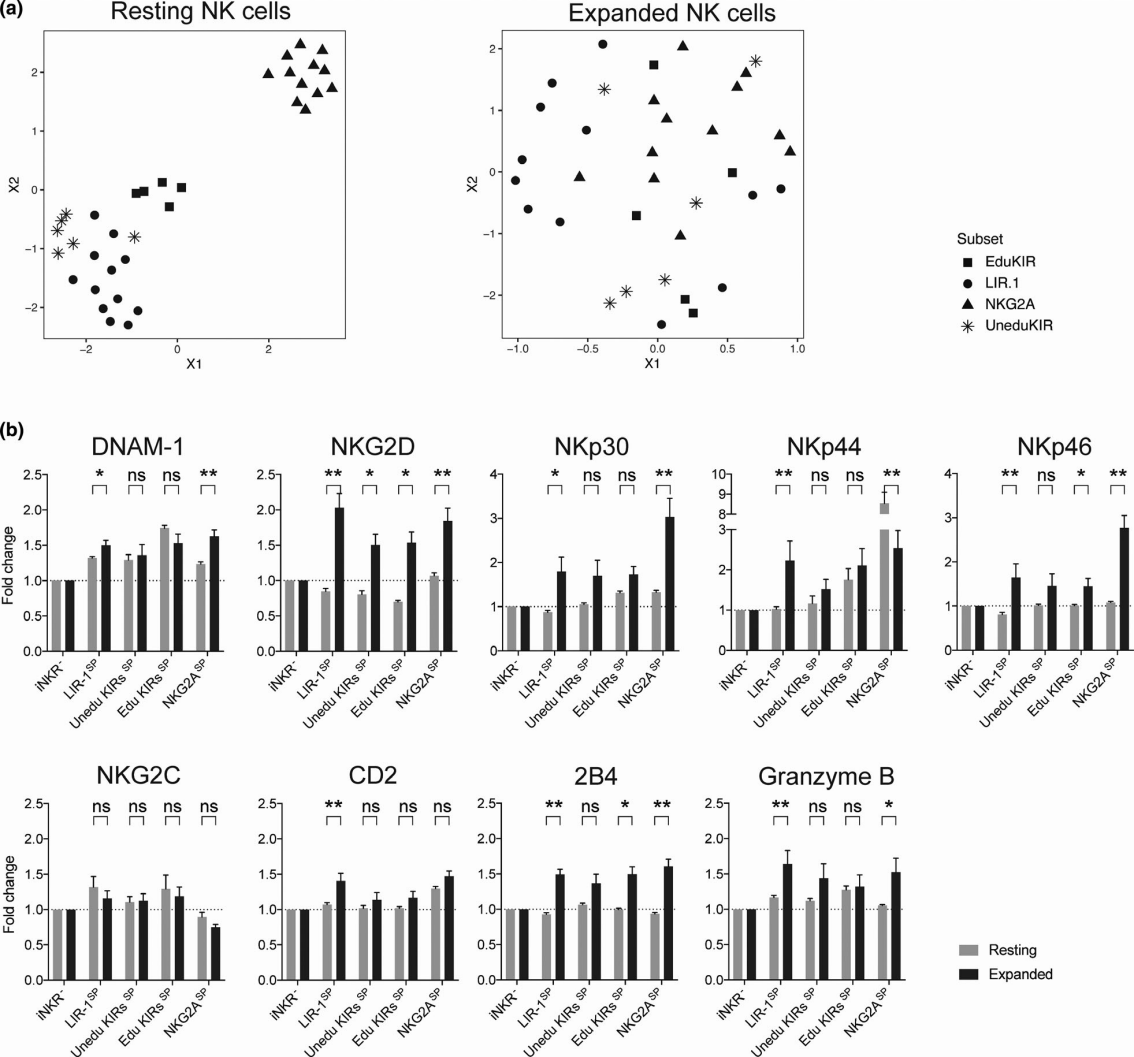
Expression of activating receptors and granzyme B by LIR‐1^SP^, uneducated KIR^SP^, educated KIR^SP^ and NKG2A^SP^ NK cell subsets compared with iNKR^‐^‐uneducated NK cells lacking these receptors. **(a)** Uniform manifold approximation and projection (UMAP) of activating receptor and granzyme B expression in GMFI on cells single positive (SP) for either a non‐educating KIR (star), educating KIR (square), LIR‐1 (circle) and the NKG2A (triangle) receptor relative to that of iNKR^‐^ NK cells within the same individual. **(b)** Relative expression as measured by GMFI of activating receptors and granzyme B on the denoted NK cell subsets compared with iNKR^‐^ NK cells (LIR‐1^SP^, *n* = 8; uneducated KIR^SP^, *n* = 7; educated KIR^SP^, *n* = 7; and NKG2A^SP^, *n* = 8). Data are shown for resting (grey) and expanded (black) NK cells from both KIR haplotype A/A and Bx donors. KIR^SP^‐positive cells have been grouped based on their educational status. Paired analysis was performed using the Wilcoxon matched‐pairs signed‐rank test. Bars show mean, and error bars represent SEM.

Confirming previous studies,^29^ *ex vivo* expansion led to an increased proportion of NKG2A^+^ NK cells (Supplementary figure 4). Next, we addressed whether the phenotype of the LIR‐1^SP^ expanded NK cell subset resembled that of educated or uneducated NK cell subsets and/or whether the increased responsiveness among LIR‐1^+^ expanded NK cells could be explained by up‐regulation of any specific activation receptor/marker. In order to make a valid comparison between resting and expanded NK cells, the expression intensity for each receptor analysed was normalised to that of each donor’s iNKR^‐^ NK cells. Thereafter, the eight‐marker data set (see Methods for details) was projected on a two‐dimensional plane with uniform manifold approximation and projection (UMAP), where each datapoint represents one subset from one individual. In contrast to resting NK cells, where the LIR‐1^SP^ subset visually grouped with the uneducated KIR^SP^ NK cell subset, whereas the KIR^SP^‐ and NKG2A^SP^‐educated NK cell subsets were in visually separate groups, no such pattern was observed within expanded NK cells (Figure 3a). Instead, the LIR‐1^SP^ expanded NK cell subset displayed a relative increased expression of all activation receptors explored compared with the corresponding subset among resting NK cells, with the exception of NKG2C (Figure 3b). In fact, several of the receptors, including NKG2D and NKp44, showed a higher relative increase in expression on the LIR‐1^SP^ expanded NK cell subset compared with any other of the expanded NK cell subsets analysed. This trend was also observed for granzyme B, an effector molecule positively linked to NK cell education.^13^, ^14^ Moreover, the expression pattern of DNAM‐1 on LIR‐1^SP^ expanded NK cells also resembled that of KIR^SP^‐ and NKG2A^SP^‐educated expanded NK cells but not that of uneducated KIR^SP^ expanded NK cells. This observation is relevant as increased DNAM‐1 expression has also been positively linked to NK cell education.^12^ Although it has not been reported previously for NKp44, a similar but more prominent pattern as for DNAM‐1 with regard to education of resting and expanded NK cells was observed.

In summary, compared with unexpanded NK cells, LIR‐1^SP^ expanded NK cells express high levels of several activation receptors/marker and have a phenotype more similar to that of NK cells educated via KIR and NKG2A, which is in stark contrast to LIR‐1^SP^ unexpanded NK cells that more resembled the phenotype of uneducated NK cells. Of note, both granzyme B and DNAM‐1 that have been positively linked to NK cell education were up‐regulated on LIR‐1^SP^ expanded NK cells.

### Detuned responsiveness by LIR‐1^+^ expanded NK cells following antibody‐mediated receptor blockade

**Figure 4:**
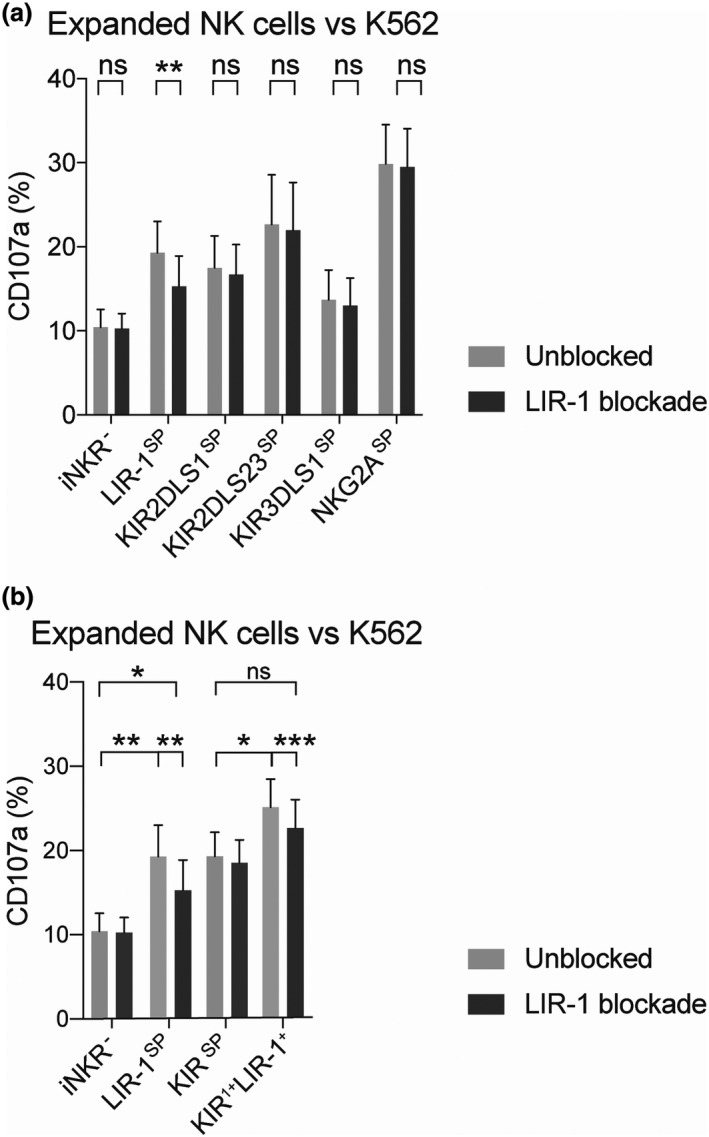
Selective detuning of the responsiveness by LIR‐1^SP^ expanded NK cells following 24‐h antibody blockade. Degranulation levels as measured by CD107a cell surface expression on expanded NK cells either pre‐blocked or not with a neutralising LIR‐1‐specific antibody for 24 h prior to co‐culture with K562. Data represent matched donors with high responsiveness by the LIR‐1 single‐positive (LIR‐1^SP^) NK cell subset against K562 and are independent of KIR education status of both KIR haplotype B/x and A/A donors. **(a)** Data presenting the degranulation by the iNKR^‐^ and the LIR‐1 and KIR SP NK subsets in the absence and presence of LIR‐1 blockade (*n* = 10, except for KIR3DL/S1^SP^ that has *n* = 6). **(b)** Pooled data comparing the responsiveness of NK cell subsets expressing either LIR‐1 or a KIR alone or co‐expressing LIR‐1 with one KIR (KIR^1+^LIR‐1^+^) compared with iNKR^‐^ (iNKR^‐^, *n* = 10; LIR‐1^SP^, *n* = 10; KIR^SP^, *n* = 24; and KIR^1+^LIR‐1^+^, *n* = 24). Paired analysis was performed using the Wilcoxon matched‐pairs signed‐rank test. Bars show mean, and error bars represent SEM.

In order to investigate the direct role of the LIR‐1 receptor in mediating increased responsiveness to target cells devoid of HLA class I, we next explored whether disruption of LIR‐1 and HLA class I interactions could detune the responsiveness of LIR‐1^+^ expanded NK cells. Similar experimental approaches have previously been used to confirm detuning of NK cells educated by KIR receptors.^30^ Following a 24‐h pre‐blockade of the LIR‐1 receptor using a specific monoclonal antibody, a selective and significant reduction of the responsiveness of the LIR‐1^SP^ NK cell subset towards K562 target cells was observed among expanded NK cells (Figure 4a). A more focused analysis on donors revealed that reduced responsiveness was also observed for NK cells co‐educated by LIR‐1 and a KIR (Figure 4b). For simplicity, the results in this study are mainly conveyed on either single expressing or co‐expression of only two receptors. However, it is important to acknowledge that a higher responsiveness could be observed among all combinations of the inhibitory receptors co‐expressing LIR‐1 and that the LIR‐1 antibody blockade was exclusively affecting LIR‐1‐positive subsets (Supplementary figure 5). The blocking effect of this monoclonal antibody has been confirmed by others,^26^ and its specificity and efficacy to block LIR‐1 was verified by us in short‐term blockade experiments using HLA class I^+^ target cells (Supplementary figure 6). Hence, these data suggest that expanded NK cells can be educated and dynamically tuned via LIR‐1 engagement.

### Co‐expression of LIR‐1 and an educating receptor on expanded NK cells is insufficient to override KIR‐ and NKG2A‐mediated inhibition by HLA class I

**Figure 5:**
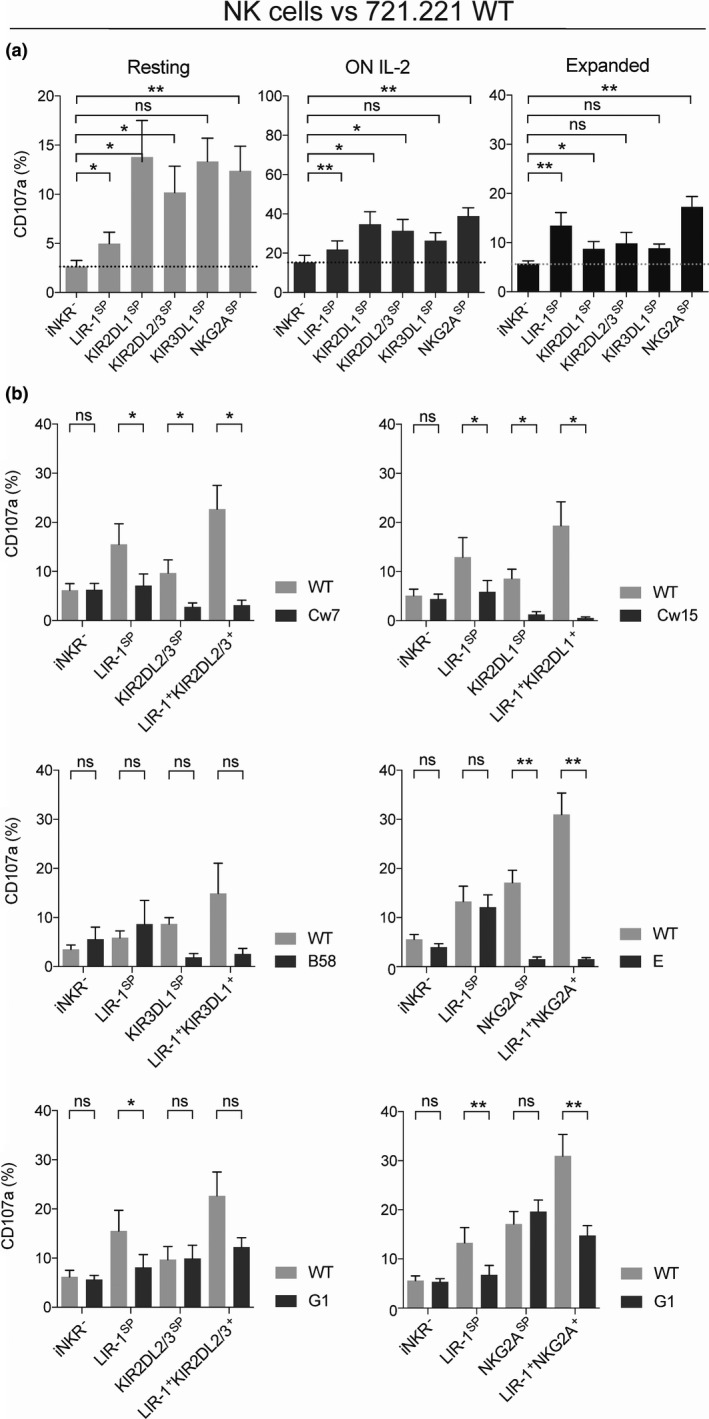
LIR‐1 co‐education with KIR and NKG2A on expanded NK cells is insufficient to overcome KIR‐ and NKG2A‐mediated inhibition. **(a)** Degranulation as measured by CD107a cell surface expression on resting, overnight (ON) IL‐2‐activated and expanded NK cells following co‐culture with HLA class I^low^ 721.221 wild‐type (WT) cells. **(b)** Degranulation by the denoted donor matched expanded NK cell subsets following co‐culture with 721.221 wild‐type (WT) or 721.221 HLA class I transfectants. Data were generated using KIR haplotype A/A donors, and only KIR‐educated NK cell subsets were selected (KIR2DL1 single positive (KIR2DL1^SP^), *n* = 7; KIR3DL1^SP^, *n* = 4; NKG2A^SP^, *n* = 10; KIR2DL2/3^SP^, *n* = 7; iNKR^‐^, *n* = 10; and LIR‐1^SP^, *n* = 10). For each graph with HLA class I transfectants, the number of donors was reduced in iNKR^‐^ and LIR‐1^SP^ subsets to resemble only matched depicted SP subsets. Paired analysis was performed using the Wilcoxon matched‐pairs signed‐rank test. Bars show mean, and error bars represent SEM.

To further address the function of LIR‐1 on expanded NK cells in the interaction with HLA class I^+^ target cells, we next performed co‐cultures with the HLA class I^low^ 721.221 wild‐type (WT) cell line and HLA class I transfectants of this cell line. As shown with K562 cells, the LIR‐1^SP^ NK cell subset had a significant increased responsiveness to 721.221 WT compared with iNKR^‐^‐uneducated NK cells (Figure 5a). The level of degranulation was similar or even higher than that of KIR‐ and NKG2A‐educated NK cells. LIR‐1^SP^ NK cells were inhibited when encountering target cells expressing HLA‐Cw7, HLA‐Cw15 and HLA‐G, but not HLA‐B58 or HLA‐E (Figure 5b). However, congruent with reports on receptor avidity,^21^ the inhibition mediated by LIR‐1 seemed weaker than that of inhibitory KIR and NKG2A receptors upon co‐culture with 721.221 cells expressing their cognate ligands. Despite LIR‐1 mediating co‐education with KIR or NKG2A receptor, enhanced responsiveness was overruled by KIR‐ and NKG2A‐mediated inhibition of the expanded NK cells. This was observed also following co‐cultures with 721.221 B58 and 721.221 E where only the KIR3DL1 receptor and the NKG2A receptor mediated inhibition, and not LIR‐1. In contrast, when co‐cultured with HLA‐G^+^ 721.221 cells, LIR‐1 was only able to mediate intermediate but not complete inhibition of NK cells co‐expressing LIR‐1 with either KIR2DL2/3 or NKG2A.

In summary, although the LIR‐1 receptor mediates co‐education of expanded NK cells this cannot override NK cell inhibition by the KIR and NKG2A receptors. Instead, co‐education can override LIR‐1‐mediated NK cell inhibition as the inhibitory capacity of this receptor seems to be weaker than that of KIR and NKG2A.

### Expanded LIR‐1^SP^ NK cells have high intrinsic ADCC capacity and a unique ability to override HLA class I‐mediated inhibition when triggered via CD16

**Figure 6:**
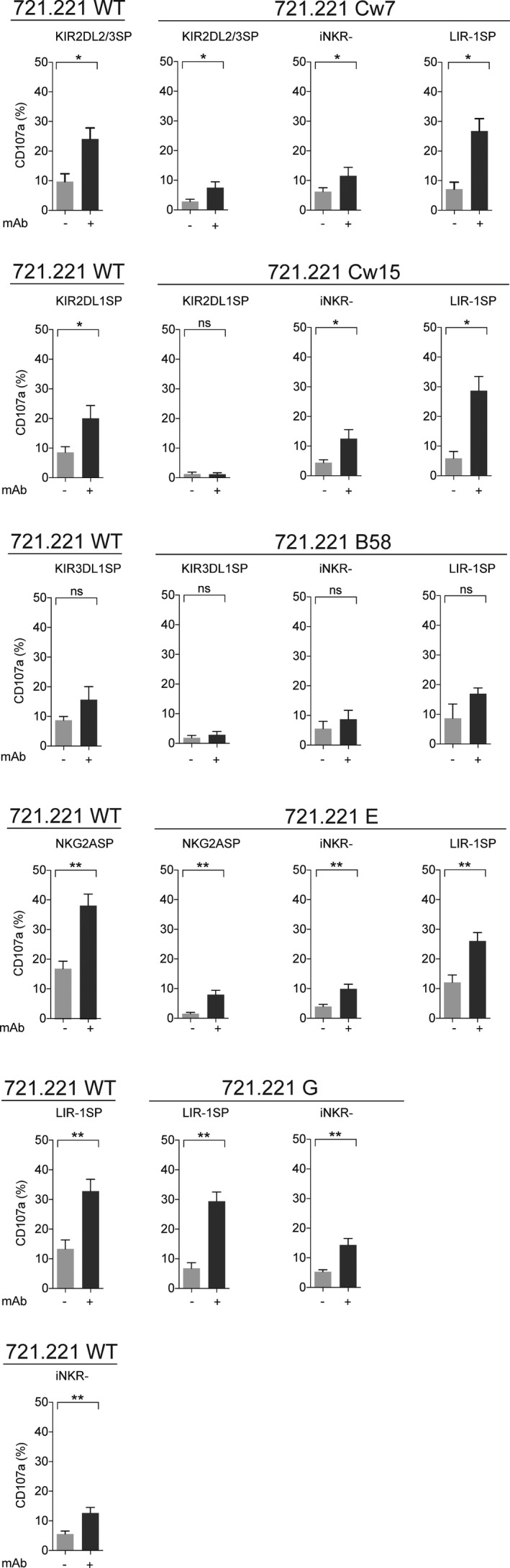
LIR‐1^SP^ expanded NK cells overcome inhibition following co‐cultures with rituximab‐coated HLA class I^+^ target cells, while this is not observed for KIR^+^ and NKG2A^+^ NK cells. Degranulation as measured by CD107a cell surface expression on the denoted expanded NK cell subsets following co‐culture with 721.221 WT or 721.221 HLA class I transfectants in the absence or presence of 10 μg mL^‐1^ rituximab. Data were generated using KIR haplotype A/A donors, and only KIR‐educated NK cell subsets were selected (NKG2A single positive (NKG2A^SP^), *n* = 10; KIR2DL1^SP^, *n* = 7; KIR2DL2/3^SP^, *n* = 7; KIR3DL1^SP^, *n* = 4; iNKR^‐^, *n* = 10; and LIR‐1^SP^, *n* = 10). For each graph, only donors having all matched depicted subsets are shown. Paired analysis was performed using the Wilcoxon matched‐pairs signed‐rank test. Bars show mean, and error bars represent SEM.

To further characterise the cytotoxic potential of LIR‐1^+^ expanded NK cells, we addressed their ability to degranulate and kill target cells via ADCC. To this end, we utilised a panel of CD20^+^ 721.221 cells that were coated with the monoclonal anti‐CD20 antibody rituximab. LIR‐1^SP^ expanded NK cells showed robust degranulation when co‐cultured with the HLA class I^low^ 721.221 WT cell line in the presence of rituximab (Figure 6), reflecting its full potential of triggering ADCC in the absence of HLA class I. Moreover, upon stimulation with rituximab‐coated 721.221 WT cells, LIR‐1^SP^ expanded NK cells induced equally potent degranulation levels as the NKG2A^SP^ NK cell subset and were significantly more potent than KIR^+^ NK cells. In contrast, the iNKR^‐^ NK cell subset showed low degranulation levels relative to the KIR^+^, NKG2A^+^ and LIR‐1^+^ NK cell subsets. Importantly, although expanded NK cells educated on KIR or NKG2A displayed robust degranulation upon CD16 engagement in the absence of HLA class I inhibition, these cells were unable to overcome self‐inhibition by HLA class I even in the presence of rituximab. In sharp contrast, despite being inhibited by HLA class I^+^ target cells in the absence of an antitumor antibody, LIR‐1^SP^ expanded NK cells triggered equally good or better degranulation following interaction with rituximab‐coated target cells regardless of the absence or presence of HLA class I on the target cells. As a result of the more potent inhibition of LIR‐1^+^ NK cells upon interaction with HLA‐Cw7, HLA‐Cw15 and HLA‐G compared with HLA‐B58 and HLA‐E, the relative increase in degranulation following triggering of the CD16 receptor was more prominent for these target cell lines.

**Figure 7:**
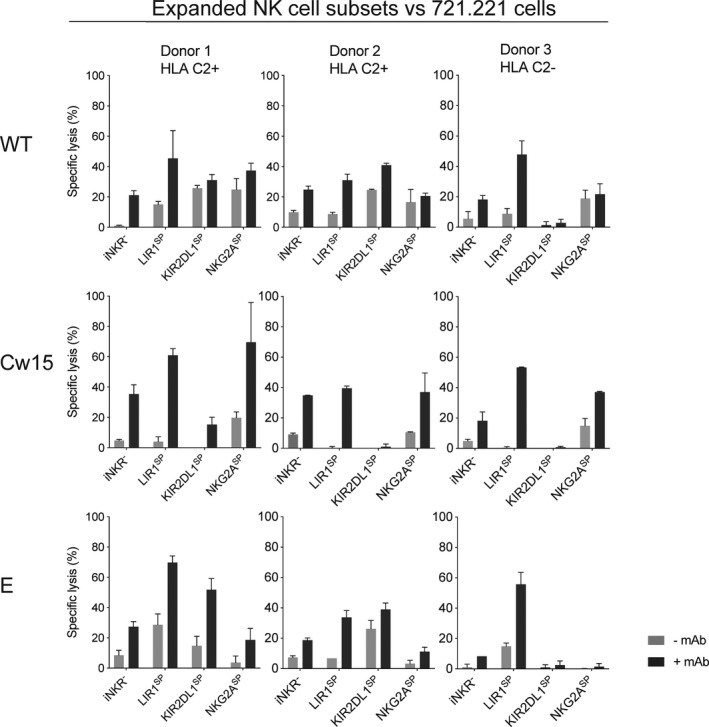
Potent antitumor cytotoxicity by LIR‐1^SP^ expanded NK cells when triggered through ADCC. Lysis of the denoted target cells as determined by ^51^Cr‐release assay following co‐cultures at an effector to target ratio of 1:1 with the denoted expanded flow cytometry‐sorted NK cell subsets in the absence or presence of 10 μg mL^‐1^ rituximab. Data are generated using three individual KIR haplotype A/A donors with the denoted KIR ligand genotype. Bars show mean of experimental triplicates, and error bars represent SD. The absence of error bars indicates that one replicate has been excluded as an outlier.

To confirm our findings with respect to NK cell degranulation, we assessed target cell killing using Chromium‐release assays with FACS‐sorted expanded NK cell subsets. LIR‐1^SP^ NK cells triggered potent ADCC as measured by target cell lysis following co‐cultures with 721.221 WT, Cw15 or E (Figure 7). KIR2DL1^SP^ and NKG2A^SP^ NK cells were inhibited upon interaction with their cognate ligand even when triggered by ADCC, whereas the LIR‐1^SP^ NK cell subset was not. Thus, these data confirmed that LIR‐1^SP^ expanded NK cells can induce potent ADCC in the absence of HLA class I and override inhibition in presence of HLA class I when triggered via CD16. Notably, CD16 expression was similar among all expanded NK cell subsets (Supplementary figure 7).

Altogether, these data emphasise that LIR‐1^+^ expanded NK cells have a potent intrinsic ADCC capacity that is not inhibited by LIR‐1 to HLA class I interactions. This unique property opens up the potential to explore this NK cell subset to target tumor cells in both autologous and allogeneic settings.

## Discussion

The concept of NK cell education was first described in humans more than a decade ago,^5^ and data published until today show a central role for the HLA class I‐binding receptors KIR and NKG2A. However, surprisingly, the LIR‐1 receptor that binds the majority of HLA class I molecules,^18^ signals via ITIMs similar to KIR and NKG2A^25^ and is expressed on a significant fraction of NK cells^31^ has not been reported to mediate NK cell education. Here, we demonstrate that LIR‐1 can mediate education of expanded, but not freshly isolated or short‐term IL‐2‐activated, NK cells. Relative to expanded iNKR^‐^ NK cells, expanded LIR‐1^SP^ NK cells displayed higher degranulation upon stimulation with tumor cell lines, as well as higher expression levels of DNAM‐1 and granzyme B. Expanded NK cells with an educating KIR co‐expressing LIR‐1 had higher degranulation levels than NK cells only expressing one of the receptors. In addition to the above, LIR‐1 blockade reduced the responsiveness of this NK cell subset, altogether suggesting that this receptor mediates NK cell education. Moreover, LIR‐1^+^ expanded NK cells mediated potent ADCC and were, in contrast to KIR^+^ and NKG2A^+^ expanded NK cells, able to override HLA class I‐mediated inhibition when triggered via CD16. Based on our findings, LIR‐1 marks a unique subset among expanded NK cells that has the intrinsic potential to reject cells that have lost HLA class I because of tumor transformation, while efficiently and selectively targeting HLA class I^+^ cells via tumor‐targeting antibodies.

It is well established that inhibitory signals from KIR and NKG2A are required to mediate and maintain education of human NK cells, yet the molecular mechanisms governing NK cell education remain poorly defined. The educational status has been linked to increased expression of DNAM‐1^12^ and granzyme B.^13^, ^14^ Recent findings indicate that the remodelling of the secretory lysosome may also be involved in determining NK cell responsiveness.^14^ However, a full understanding of what network of intracellular signalling pathways and other factors that regulate NK cell education is lacking today. Based on current knowledge, one may speculate that despite LIR‐1 signals via ITIMs similar to KIR and NKG2A,^32^, ^33^ the weaker NK cell inhibition mediated by LIR‐1 compared with KIR and NKG2A is insufficient to trigger efficient NK cell education of unexpanded NK cells.^21^ The intracellular signalling machinery may be altered in NK cells following expansion, facilitating NK cell education via LIR‐1. Nonetheless, LIR‐1^+^ expanded NK cells also show less potent inhibition compared with KIR^+^ and NKG2A^+^ expanded NK cells. Another non‐mutually exclusive explanation is the altered expression of key activating receptors observed on LIR‐1^SP^ NK cells following expansion, where the phenotype of expanded LIR‐1^SP^ NK cells more closely resembled that of KIR‐educated NK cells than that of unexpanded LIR‐1^SP^ NK cells. This was most prominent for DNAM‐1 and NKp44, while a significant increase in granzyme B and NKG2D was also observed for this subset.

Under normal conditions, it has been suggested that LIR‐1 regulates NK cells in the fetal–maternal interface by preventing the rejection of the foetus by inhibiting lysis of trophoblasts by maternal NK cells via interactions with HLA‐G.^23^ We speculate that LIR‐1 on NK cells may also have a prominent role in the context of inflammation, as LIR‐1 is acquired in later stages of NK cell maturation^34^ and is expressed on adaptive NK cells. Following an immune response with NK cell proliferation at the inflamed site, LIR‐1 may be an important checkpoint‐like receptor controlling NK cell reactivity to normal HLA class I^+^ self‐cells, while being reactive to transforming cells losing HLA class I or to which humoral immunity has been raised. Congruently, relative to healthy controls, increased frequencies of NK cells expressing LIR‐1 have been observed in patients with malignancies such as colorectal cancer, triple‐negative breast cancer (TNBC) and acute myeloid leukaemia,^35^, ^36^, ^37^ who often have sustained systemic inflammation. As a proof for LIR‐1 being relevant in targeting of cancer cells, investigators have shown that LIR‐1 blockade augments cetuximab‐mediated ADCC against TNBC.^35^ Moreover, LIR‐1 blockade improves chronic lymphocytic leukaemia targeting by lenalidomide‐exposed NK cells^38^ and triggers NK cell cytotoxicity against primary leukaemic blasts.^26^ Based on our finding and the literature, LIR‐1 seems to have a role in controlling NK cells in settings of cancer although further studies are needed to dissect the exact role of the LIR‐1 receptor on endogenous NK cells and its ability to naturally mediate education *in vivo*.

An important and novel finding in our study was that LIR‐1 marked an expanded NK cell subset with potent ADCC capacity and the unique ability to override inhibition by HLA class I upon CD16 stimulation. The latter was in sharp contrast to KIR^+^ and NKG2A^+^ expanded NK cells that were completely inhibited by their cognate ligand in the context of ADCC. The finding that ADCC can override LIR‐1‐mediated inhibition contradicts previous data by Colonna *et al*.^39^; however, their data were generated in reverse ADCC experiments with unexpanded NK cells, while our results were obtained from co‐cultures assays with expanded NK cells.

The clinical implications of our discovery are intriguing for several reasons. First, the potent LIR‐1^+^ expanded NK cell subset is inhibited by most HLA class I molecules and should therefore be considered safe to adoptively transfer across HLA class I barriers. Second, the subset should maintain its responsiveness post‐infusion not only in an autologous setting, but also in an allogeneic setting, which differs from KIR where a KIR‐KIR ligand match is required for this to occur. Third, as this subset, in contrast to KIR^+^ and NKG2A^+^ expanded NK cells, has potent ADCC capacity and at the same time can overcome inhibition by HLA class I when triggered by CD16, LIR‐1^+^ expanded NK cells are attractive to explore in cancer immunotherapy protocols involving antitumor antibodies in both autologous and allogeneic settings. Finally, given that these cells acquire their features following *ex vivo* expansion, high numbers of NK cells with these properties could be utilised for multiple rounds of infusion. Overall, these qualities make LIR‐1^+^ expanded NK cells an attractive NK cell subset with clear therapeutic implications, including the use as an off‐the‐shelf product. Future investigations will hopefully address the potential of this subset in clinical cancer immunotherapy. However, prior to that, ways to selectively expand or by other means produce a large amount of these cells would facilitate the clinical translation as with the current expansion protocol used in this study, only a smaller fraction of the expanded NK cells are LIR‐1^SP^.

Our study has strengths and limitations. While data were generated in a controlled experimental setting with well‐characterised donors and target cells along with gold standard methods to address NK cell responsiveness, the study does not dissect what intracellular signalling pathways that are involved in mediating increased responsiveness by LIR‐1^SP^ expanded NK cells. Moreover, our understanding of the underlying mechanisms that govern NK cell education remains mechanistically largely inexplicable. In the current work, we have relied our conclusions on analyses showing that expanded NK cells expressing LIR‐1 have increased responsiveness compared with the iNKR^‐^ subset and that LIR‐1 shows signs of co‐educating expanded NK cells with KIR. In parallel, we have also based our conclusions on experiments where antibody masking of LIR‐1 for 24 h, thereby preventing its binding to HLA class I, reduces the potency of expanded NK cells expressing LIR‐1 indicative of detuning. Although the antibody showed LIR‐1‐specific reduction of degranulation levels on expanded NK cells co‐cultured with HLA class I‐deficient K562 cells, while it also could be used to revoke HLA class I inhibition of non‐expanded NK cells expressing LIR‐1 supporting neutralising actions, it would have been desirable to also establish detuning in a complementary mechanistic setting in order to completely confirm remodelling of the NK cell education status while ruling out indirect effects of the antibody approach *per se*. Furthermore, although we observed increased responsiveness in the vast majority of donors from which we expanded NK cells, the degree of responsiveness varied and the factors determining the level of responsiveness remain unclear. In future efforts to translate these findings to the clinic, the impact of different LIR‐1 alleles and polymorphisms,^40^, ^41^ as well as what transcriptional variants that are expressed,^42^, ^43^ also needs consideration as this can affect the avidity of HLA class I binding. Although LIR‐1 binds the conserved β_2_m and α3 regions of the HLA class I molecule,^20^, ^21^ it has different avidities for different HLA class I molecules.^44^ Nevertheless, the numerous polymorphisms of LIR‐1^40^ are likely to be more of a key factor dictating the avidity and thereby education level induced by LIR‐1 on expanded NK cells, especially given that several HLA class I alleles are expressed within a given individual. Thus, sizeable cohorts are needed to fully address these questions and are beyond this first report.

In addition to the above, animal studies are warranted to establish whether LIR‐1 mediates NK cell education *in vivo* and to address the potential of the LIR‐1^+^ NK cell subset in cancer immunotherapy. However, preclinical animal models are in this case associated with several challenges. In contrast to the KIR ortholog Ly49 that is expressed on murine NK cells and that has been used to delineate NK cell education *in vivo*, the murine LIR‐1 ortholog PIR‐B is not expressed by murine NK cells hindering such studies.^45^ Whether LIR‐1‐mediated education of the expanded human NK cells is maintained following infusion would therefore have to be investigated using HLA class I transgenic or humanised mice. Such approach could be complemented with *ex vivo* correlative studies performed as part of clinical trials exploring adoptive transfer of *ex vivo* expanded NK cells. Although the therapeutic potential of LIR‐1^+^ expanded human NK cells could be evaluated using immune‐deficient mice inoculated with human tumor cells, this may not be the optimal model as LIR‐1‐mediated education may not be maintained. An ideal model would therefore be humanised mice; however, establishing tumors in immune‐competent animals has historically been difficult, hence making such approach less feasible.

In conclusion, our data reveal for the first time that the inhibitory HLA class I‐binding receptor LIR‐1 can mediate education of NK cells. This was exclusively observed among expanded but not for resting and ON IL‐2‐activated NK cells. Our data also show that although LIR‐1^+^ expanded NK cells are inhibited by HLA class I^+^ target cells, ADCC triggers potent activation of these NK cells that override inhibition by HLA class I, which is in sharp contrast to KIR and NKG2A. Given that LIR‐1^+^ expanded NK cells are inhibited by most HLA class I molecules and thereby also educated by these molecules, LIR‐1^+^ expanded NK cells exhibit the potential to be used in both autologous and allogeneic settings. To further detail the potential of this subset for clinical therapy, additional studies that include patient samples and translational animal models are needed.

## Methods

### Isolation and expansion of NK cells

Healthy donor peripheral blood mononuclear cells (PBMCs) were collected by high‐density gradient centrifugation following informed consent (protocol 99‐H‐0050 (NIH) and 2006/229‐31/3 (KI)). NK cells were isolated from PBMCs using magnetic bead separation (NK cell isolation kit or the CD3 depletion kit followed by the CD56‐positive selection kit, all from Miltenyi, Bergisch Gladbach, Germany) according to the manufacturer’s instructions. Resting and overnight (ON) IL‐2‐activated NK cells were cultured in RPMI 1640 (Gibco, Waltham, MA, USA) media containing 10% fetal bovine serum (FBS) (Gibco) supplemented with or without 1000 IU mL^‐1^ IL‐2 (Chiron, Emeryville, CA, USA/PeproTech, Rocky Hill, NJ, USA). NK cells were expanded *ex vivo* according to Berg *et al*.^29^ In brief, isolated NK cells were mixed with 100 Gy‐irradiated SMI‐LCL feeder cells at a ratio of 1:20 in 15 mL of expansion media containing X‐VIVO 20 (Lonza, Walkersville, MD, USA) cell culture medium containing 10% heat‐inactivated human AB serum (Invitrogen), 2mM GlutaMAX‐1 (Gibco) and 500 IU mL^‐1^ IL‐2 in standing 75‐cm^2^ cell culture flasks in an incubator set at 6.5% CO_2_ and 37°C. Fresh expansion media were supplied first after 4 days, after which cells were subcultured every 2–3 days to 0.5 million cells mL^‐1^ after reaching concentrations over 1 million cells mL^‐1^. Cells were harvested and used after 13–15 days of expansion. In selected experiments, NK cells were expanded without feeder cells using the same expansion media, although cultured at greater density.

### KIR/KIR ligand genotyping

In order to assess the NK cell education status of specific donors, genomic DNA was extracted using the DNeasy Blood & Tissue Kit (Qiagen, Venlo, the Netherlands) and genotyping was performed by PCR using Olerup SSP KIR Genotyping and KIR HLA Ligand Kits (Olerup SSP, Stockholm, Sweden) according to the manufacturers’ instructions.

### Cell lines and reagents

All cell lines used were cultured in RPMI 1640 media (Gibco) containing 10% FBS (Gibco). The lymphoblastoid cell line (LCL) SMI‐LCL was established following Epstein–Barr virus (EBV) transformation of healthy donor B cells by Dr Childs’ group at the NIH. The EBV‐LCL 721.221 cells were a kind gift from Dr Parham, Stanford, USA. The 721.221 Cw7 and E transfectants were cultured in media supplemented with Hygromycin B Gold (Invivogen, San Diego, CA, USA) at a final concentration of 0.3 mg mL^‐1^. The 721.221 B58, G and Cw15 transfectants were cultured in media supplemented with G‐418 (Sigma‐Aldrich, St Louis, MO, USA) at a final concentration of 0.5 mg mL^‐1^. The K562, MOLM‐14 and HL‐60 cell lines were obtained from ATCC (Manassas, VA, USA).

### Flow cytometry

Cells were labelled with the following antibodies and reagents for flow cytometry: anti‐KIR2DL1‐FITC (143211) from R&D Systems (Minneapolis, MN, USA); anti‐NKG2C‐FITC (REA205) from Miltenyi; anti‐KIR2DL/DS2/3‐PECy5.5 (GL183), anti‐NKG2A‐PE and anti‐NKG2A‐PECy7 (Z199), and anti‐KIR2DL/DS1‐PE (EB6) from Beckman Coulter (Brea, CA, USA); anti‐KIR3DL1‐V421 and anti‐KIR3DL1‐AF700 (Dx9), anti‐NKG2D‐APC‐Cy7 (1D11), anti‐2B4‐PE/Dazzle™ 594 (C1.7), anti‐CD107a‐PB, anti‐CD107a‐BV605 and anti‐CD107a‐BV785 (H4A3), anti‐CD16‐PB (3G8), anti‐HLA class I‐PE (W6/32) and anti‐HLA‐E‐APC (3D12) from Biolegend (San Diego, CA, USA); anti‐CD3‐V500 (UCHT1), anti‐CD16‐BV650 (3G8), anti‐CD57‐BV605 (NK‐1), anti‐CD2‐BV711 (RPA‐2.10), anti‐DNAM‐1‐BV786 (Dx11), anti‐granzyme B‐PE‐CF595 (Gb11), anti‐NKp44‐BV650 (p44‐8), anti‐NKp30‐BV711 (p30‐15), anti‐NKp46‐BV786 (9E2/NKp46), anti‐CD56‐PECy7 and anti‐CD56‐BUV737 (NCAM16.2), and BD^TM^ CompBeads from BD Biosciences (San Jose, CA, USA); and fixable dead cell marker LIVE/DEAD Aqua and anti‐LIR‐1‐APC (HP‐F1) (also LifeSpan Biosciences Inc., Seattle, WA, USA) from Invitrogen eBiosciences (Waltham, MA, USA).

In brief, cells were mixed and incubated with antibodies for extracellular markers for 15–30 min at 4°C in the dark. Cells were then washed three times in FACS buffer containing 2 mm EDTA and 2% FBS in phosphate‐buffered saline (PBS) (Gibco) and finally fixed in 1% paraformaldehyde (Polysciences, Warrington, PA, USA) for 15 min at 4°C. For intracellular staining, cells were permeabilised after fixation for 6–8 min using 0.1% Triton X‐100 (Sigma‐Aldrich) in PBS at room temperature (RT), followed by washing in FACS buffer before being mixed and incubated with antibodies for intracellular markers for 30 min at RT in the dark. Finally, the cells were washed and resuspended in FACS buffer prior to acquisition on an LSRFortessa™ (BD Biosciences). Cell sorting was performed on unfixed cells, stained for extracellular markers and using FACS buffer with addition of Pen‐Strep (Gibco) and then acquired on an FACSAria™ Fusion Cytometer (BD Biosciences). Data were analysed using the FlowJo software (BD Biosciences).

### Degranulation assay

NK cells and target cells were co‐cultured at a ratio of 1:1 for 1 h at 37°C and 5% CO_2_. Following co‐culture, cells were stained with fluorescently conjugated antibodies for CD107a expression and markers for the identification of specific NK cell subsets. A condition without target cells was used as a negative control. In order to assess ADCC, 10 μg mL^‐1^ of rituximab (Roche, Basel, Switzerland) was added to the co‐cultures. Whereas data for resting and ON IL‐2‐activated NK cells were based on the CD56^dim^ NK cell subset, data for expanded NK cells were generated from the whole CD56^+^ population as the CD56^bright^ and CD56^dim^ NK cell populations cannot be discriminated following *ex vivo* expansion. A Boolean gating strategy was applied for subset analysis. A representative gating strategy on NK cells without target cells can be found in Supplementary figure 8, corresponding to the same donor that NK cell degranulation is shown for in Figure 1a.

### Antibody blockade of LIR‐1

The LIR‐1 antibody MAB20172 clone 292319 (R&D Systems), binding to a different epitope on the LIR‐1 molecule compared with the HP‐F1 clone used for phenotyping, was used to block the receptor. NK cells were exposed to 10 μg mL^‐1^ either 15 min or 24 h prior to a degranulation assay to address its short‐ and long‐term effect, respectively.

### ^51^Chromium‐release assay

Immediately after FACS‐based cell sorting, NK cell subsets were co‐cultured at a ratio of 1:1 with ^51^Cr‐labelled K562 or 721.221 cells in the absence or presence of 10 μg mL^‐1^ rituximab (Roche) in a final volume of 200 μL in 96‐well plates at 37°C and 5% CO_2_. Each condition was run in triplicates. Target cells cultured in media alone or in 3% SDS were used for assessing spontaneous and maximum ^51^Cr release, respectively. After 4 h, the supernatant was harvested and transferred to a Luma plate. Counts were measured using a WIZARD2 Automatic Gamma Counter (PerkinElmer, Waltham, MA, USA), and specific target lysis was calculated using the formula 100x ((NK cell‐induced ^51^Cr‐release – spontaneous ^51^Cr‐release)/(maximum ^51^Cr‐release – spontaneous ^51^Cr‐release)). Individual replicates with more than two standard deviations from the two remaining replicates were excluded as outliers.

### Uniform manifold approximation and projection (UMAP) analysis

For the non‐expanded and expanded cells, the following procedure was performed separately: First, the geometric mean fluorescence intensity (GMFI) values for 2B4, NKp30, NKp44, NKp46, NKG2D, granzyme B, DNAM‐1, and CD2 for NK cell subsets being NKG2A^SP^, LIR‐1^SP^ or single positive for either a non‐educating or educating KIR were normalised to the GMFI of the iNKR^–^ subset for the same individual. Then, to tune the relative influence on the UMAP of the different markers, the data were robustly scaled and centred, so that the 5th and 95th percentile were set to −0.5 and 0.5, respectively. Then, an UMAP was constructed, using the uwot package^46^ in R.^47^

### Statistical analysis

Statistical analyses were performed using Prism (GraphPad Software Inc., San Diego, CA, USA). The Wilcoxon matched‐pairs signed‐rank test was used to assess significance in paired non‐parametric data sets. The Mann–Whitney *U*‐test was used for unpaired non‐parametric data sets. Student’s *t*‐tests were used to compare data that could be assumed to be parametrical. Significance tests performed on fold change data were compared with a hypothetical value of 1. *P* < 0.05 *, *P* < 0.01 **, *P* < 0.001 *** and *P* < 0.0001**** were considered statistically significant, and ns denotes not significant.

## Conflict of interest

The authors declare no competing interest related to this work.

## Author contributions

**Caroline Leijonhufvud:** Conceptualization; Data curation; Formal analysis; Investigation; Methodology; Visualization; Writing‐original draft; Writing‐review & editing. **Robert Reger:** Data curation; Investigation; Methodology; Writing‐review & editing. **Filip Segerberg:** Data curation; Investigation; Methodology; Visualization; Writing‐review & editing. **Jakob Theorell:** Formal analysis; Methodology; Visualization; Writing‐review & editing. **Heinrich Schlums:** Methodology; Writing‐review & editing. **Yenan Bryceson:** Resources; Writing‐review & editing. **Richard William Childs:** Conceptualization; Resources; Supervision; Writing‐review & editing. **Mattias Carlsten:** Conceptualization; Formal analysis; Funding acquisition; Methodology; Resources; Supervision; Writing‐original draft; Writing‐review & editing.
