# Supplementary material for: LIR‐1 educates expanded human NK cells and defines a unique antitumor NK cell subset with potent antibody‐dependent cellular cytotoxicity
Source: Clin Transl Immunology. 2021 Oct 5;10(10):e1346. doi: 10.1002/cti2.1346 (PMC8491220; doi:10.1002/cti2.1346)
Supplement: Supplementary file 1 [file CTI2-10-e1346-s001.pdf]

Supplementary figure 1. Leijonhufvud et al.

Expanded NK cells vs K562

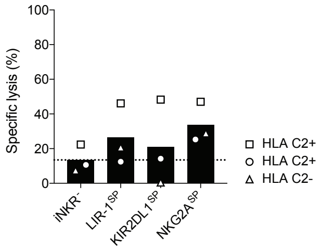


**Supplementary figure 1. $^{51}$Cr-release assays show robust cytotoxicity by LIR-1$^{SP}$ expanded NK cells relative to uneducated NK cell subsets.** Lysis of K562 cells by flow cytometry-sorted subsets of expanded NK cell either lacking all of the investigated inhibitory receptors (iNKR$^{-}$) or single positive (SP) for LIR-1, KIR2DL1 or NKG2A at E:T ratios of 1:1. Square, circle (educated on KIR2DL1) and triangle (not educated on KIR2DL1) represent mean of triplicates from 3 individual KIR haplotype A/A donors having different HLA-C genotype and NK cell education status via KIR2DL1. Bars show mean.

Supplementary figure 2. Leijonhufvud et al.

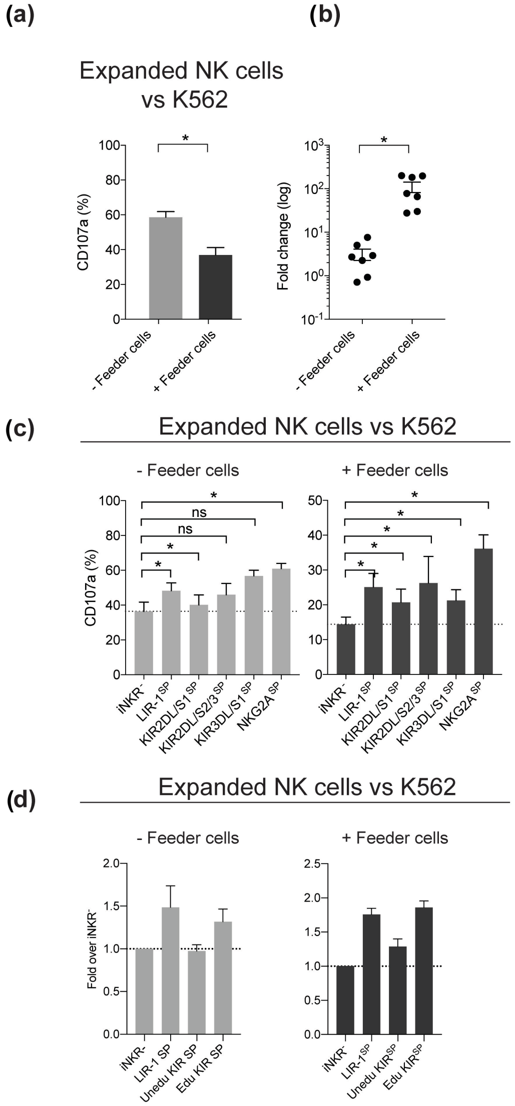


**Supplementary figure 2. NK cell expansions with and without feeder cells both yield higher responsiveness of LIR-1$^{SP}$ NK cells compared to uneducated iNKR$^{-}$ NK cells. (a)** Degranulation as measured by CD107a on bulk NK cells against K562. **(b)** Expansion rates in fold changed over starting cell number. **(c)** Degranulation against K562 by the denoted NK cell subsets independently of KIR education status among NK cells expanded with and without feeder cells, n=7. D) Degranulation against K562 by the denoted NK cell subsets sub-grouped according to the genomic presence and absence of inhibitory receptors and their cognate HLA class I ligands among NK cells expanded with and without feeder cells (5 donors in total that could be divided into; LIR-1 single positive (LIR-1$^{SP}$) n=5, uneducated KIR$^{SP}$ n=6, educated KIR$^{SP}$ n=5, NKG2A$^{SP}$ n=5). The data was generated from both KIR haplotype A/A and B/x donors . Paired analysis was performed using Wilcoxon matched-pairs signed-rank test. Bars show mean and error bars represent SEM.

Expanded NK cells vs K562

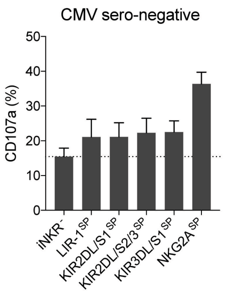


**Supplementary figure 3. Increased responsiveness by LIR-1$^{SP}$ NK cells among CMV sero-negative individuals.** Degranulation against K562 cells by expanded NK cell subset expressing only the denoted receptor (single positive; SP) or lacking all of the denoted receptors (iNKR$^{-}$). NK cells from blood donors (n=5) were used that tested negative for Cytomegalovirus (CMV) IgG in the clinical routine screening protocol upon blood collection at the Karolinska University Hospital Laboratory. The data was generated from both KIR haplotype A/A and B/x donors and is independent of KIR education status. Bars show mean and error bars represent SEM.

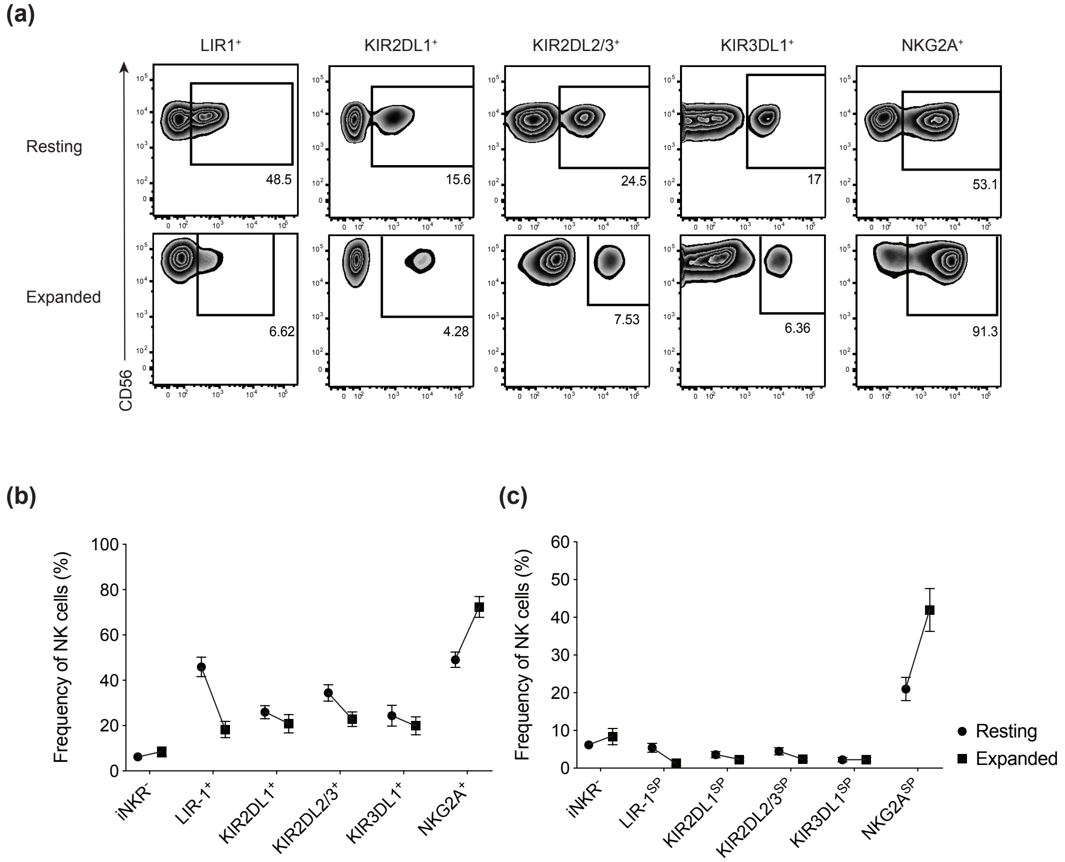


**Supplementary figure 4. Inhibitory receptor phenotype expression on resting and expanded NK cells. (a)** Zebra plots from one representative donor showing the expression of the denoted inhibitory receptor. **(b)** Frequency of NK cells expressing each denoted inhibitory receptor as well the frequency of NK cells lacking all of the investigated inhibitory receptors (iNKR$^-$). **(c)** Frequency of NK cells single positive (SP) for each denoted inhibitory receptor and the iNKR$^-$ NK cell subset. Data represent mean of matched resting (circles) and expanded (squares) KIR haplotype A/A donors (n=10 except for KIR3DL1 n=9). Error bars represent SEM.

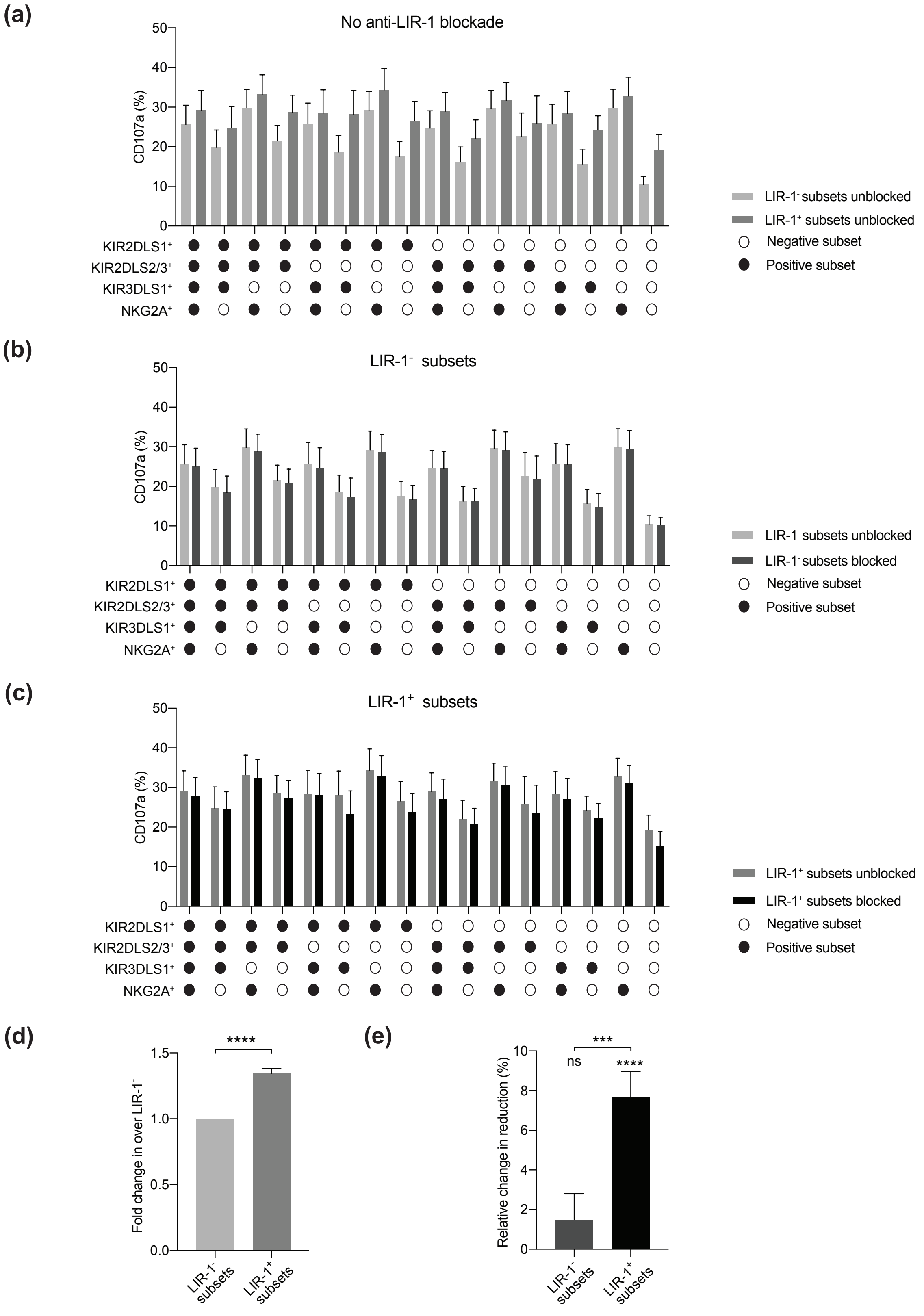


**Supplementary figure 5. Global and selective effect on LIR-1 co-expressing expanded NK cell subsets' responsiveness following 24-hour antibody blockade**. Degranulation levels as measured by CD107a cell surface expression on expanded NK cells either pre-blocked or not with a neutralizing LIR-1-specific antibody for 24 hours prior to co-culture with K562. *Legend continues on next page.*

**Supplementary figure 5.** *Continued.* Data represent matched donors with high responsiveness by the LIR-1 single positive (LIR-1$^{SP}$) NK cell subset against K562 and is independent of KIR education status of both KIR haplotype B/x and A/A donors. Data show the degranulation by all 32 possible inhibitory receptor subsets with sufficient cell numbers, from 5-10 donors depending on subset frequency in separate donors in, **a)** unblocked NK cell subsets with or without LIR-1 co-expression (LIR-1$^{+}$), **b)** LIR-1$^{-}$ subsets with or without LIR-1 antibody blockade or **c)** LIR-1$^{+}$ subsets with or without LIR-1 antibody blockade. Paired and pooled subsets (n=125) from a) are shown in **d)** by fold change of unblocked LIR-1$^{+}$ divided by LIR-1$^{-}$ NK cell subsets and compared to a hypothetical value of 1 using One-sample *t*-test. Paired and pooled subsets (n=125) from b) and c) are show in **e)** as relative percental change in reduction from LIR-1 antibody blockade on LIR-1$^{-}$ and LIR-1$^{+}$ subsets respectively calculated by $\left(\frac{\mathrm{CD107a}^{\mathrm{Unblocked}} - \mathrm{CD107a}^{\mathrm{Blocked}}}{\mathrm{CD107a}^{\mathrm{Unblocked}}}\right) * 100$ and compared to each other by a paired Student *t*-test or alone against a hypothetical value of 0 using One-sample *t*-test. Bars represent mean and error bars represent SEM.

Supplementary figure 6. Leijonhufvud et al.

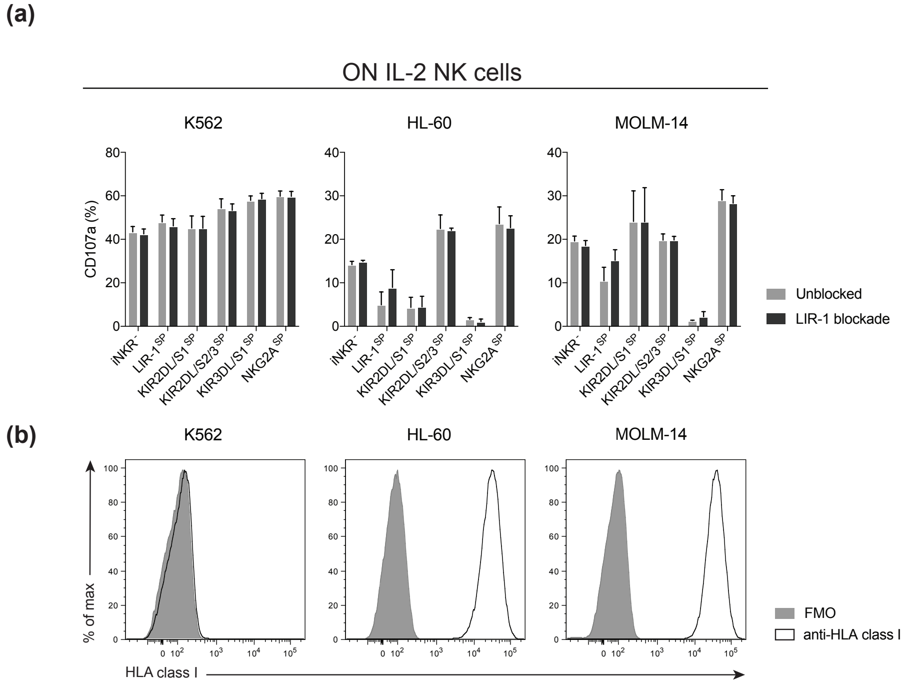


**Supplementary figure 6. Short-term antibody blockade of LIR-1 triggers augmented targeting of HLA class I$^{+}$ tumor cell lines by the LIR-1$^{SP}$ NK cell subset. (a)** Degranulation by the denoted over-night (ON) IL-2-activated NK cell subsets either unblocked or pre-blocked for 15 minutes with a LIR-1-specific antibody prior to being co-cultured with K562 (n=6), HL-60 (n=3), MOLM-14 (n=3) target cells. The data was generated from both KIR haplotype A/A and B/x donors and is independent of KIR education status. SP, single positive. iNKR$^{-}$, NK cell inhibitory receptor negative. **(b)** HLA class I expression on K562, HL-60 and MOLM-14 target cells. FMO, fluorescence minus one. Bars show mean and error bars represent SEM.

Supplementary figure 7. Leijonhufvud et al.

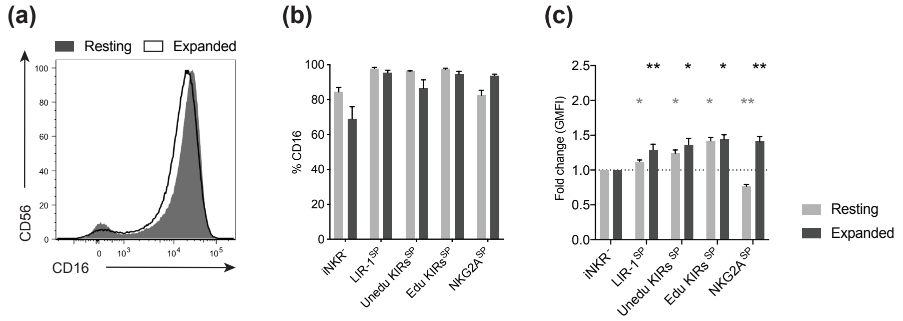


**Supplementary figure 7. Expression of CD16 by resting and expanded NK cell subsets. (a)** A representative example of CD16 expression on resting (filled) and expanded (solid line) NK cells from one donor. **(b)** Proportion of $CD16^{+}$ NK cells on the denoted NK cell subsets. $iNKR^{-}$, NK cell inhibitory receptor negative. **(c)** Relative expression as measured by GMFI of CD16 on the denoted NK cell subsets compared to $iNKR^{-}$ NK cells. Data are shown for resting (grey) and expanded (black) NK cells from both KIR haplotype A/A and Bx donors. $KIR^{SP}$ NK cells have been pooled based on their educational status. $LIR1^{SP}$ n=8, uneducated $KIR^{SP}$ n=7, educated $KIR^{SP}$ n=7, $NKG2A^{SP}$ n=8. Individual groups were compared to $iNKR^{-}$ using Wilcoxon signed rank test and comparing to a hypothetical value of 1. Bars show mean and error bars represent SEM.

Supplementary figure 8. Leijonhufvud et al.

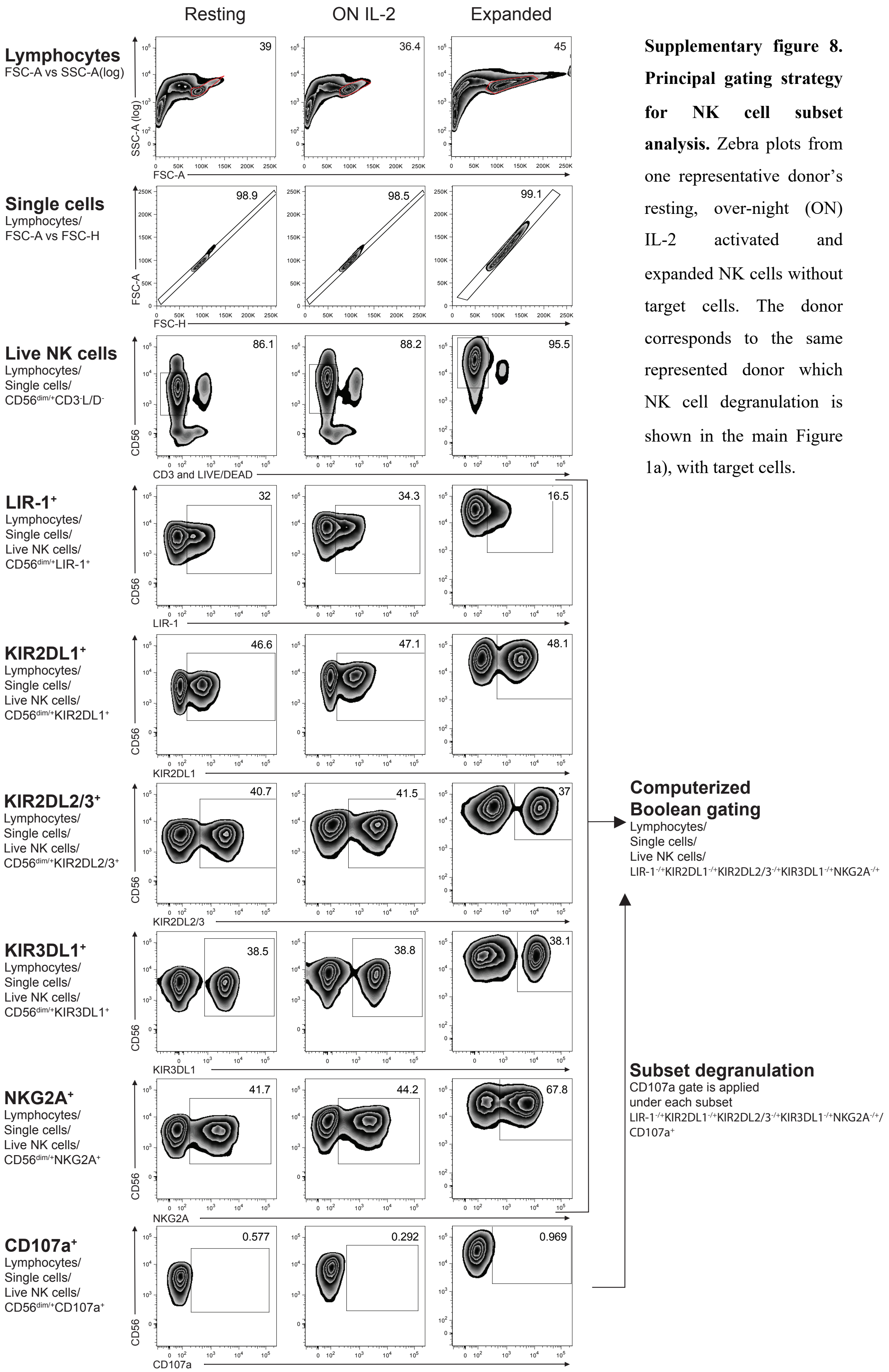


**Supplementary figure 8. Principal gating strategy for NK cell subset analysis.** Zebra plots from one representative donor's resting, over-night (ON) IL-2 activated and expanded NK cells without target cells. The donor corresponds to the same represented donor which NK cell degranulation is shown in the main Figure 1a), with target cells.
